# Devising Solutions for COVID-19 Fear in Type 2 Diabetes Mellitus

**DOI:** 10.1177/00302228211029142

**Published:** 2021-06-26

**Authors:** Megha Nataraj

**Affiliations:** Department of Physiotherapy, Centre for Diabetic Foot Care & Research, Manipal College of Health Professions, Manipal Academy of Higher Education, India

**Keywords:** coronavirus, physical activity, health, pandemic, fear

## Abstract

This letter to the editor’s article highlights the role of incorporating physical activity as a critical component in addressing fear among type 2 diabetes mellitus individuals during the COVID-19 pandemic.

With great interest, I read the article by Kaplan Serin and Bülbüloğlu (2021) on the effects of attitude to death on self-management among patients with type 2 diabetes mellitus during the present COVID-19 pandemic. The authors discuss the complications of neuropathy (20.4%), nephropathy (14.6%) and cardiovascular disease (12.6%) to be the highest among their study group. Furthermore, they report COVID-19 fear to be statistically higher among the diabetic patients with neuropathy and nephropathy. A vast majority of their study participants (70.9%) did not engage in any form of physical activity or exercise.

I would discuss hereafter that the common link between emotions, diabetes, hypertension, cardiovascular disease, nephropathy, and neuropathy is the overstimulation of the sympathetic nervous system. It is critical to understand the source of rising emotions. Every emotional experience is a response to a given stimulus. In the present situation, our constant stimulation is the chronic threat of COVID-19, which arouses a fight, flight response reaction in the body. An emotional upsurge is thereby experienced as a result of uncertainty and lack of control over our lives. Patients with diabetes mellitus fear their low levels of immunity which would increase their susceptibility to contracting COVID-19. They fear going out of stock of necessary medications due to worldwide lockdown restrictions.

Sympathetic arousal can be dealt with effectively by incorporating exercise or bouts of physical activity in an individual’s daily routine. The positive mental health benefits of physical activity have been well explained across psychological mechanisms such as the distraction hypothesis, self-efficacy theory, mastery theory, and social interaction hypothesis. These mechanisms produce an anti-depressive effect on the mind as explained in mono-amine hypothesis, endorphin hypothesis and the thermo genic model. Improved blood circulation in different regions of the brain is regulated through the hypothalamic-pituitary axis when an individual engages in exercise.

The role of leisure time physical activity and domestic physical activity is lately gaining importance for its mental health benefits. Apart from the locomotive nature, the skeletal muscles have a secretary function (Pedersen et al., 2013) through the release of myokines, which are responsible for the regulation of fat and glucose metabolism in the body. Physical inactivity reverses these beneficial effects and eventually contributes to the altered regulatory mechanism of glucose, increasing the risk of developing non-communicable diseases. Improvement in lifestyle can be achieved through increasing the exposure to non-exercise activity thermogenesis (Levine, 2007). A large portion of the total daily energy expended by an individual is spent in light-intensity activities. As domestic and leisure-time physical activity (Holstila et al., 2017) are of a light-intensity nature, by increasing the time/duration spent on doing them, some beneficial effects may be observed on both physical and mental health.

The Physical Activity Guidelines (Scientific Report, 2020; Segar et al., 2020) recommend and encourage participation in different physical activity domains. However, physical activity's therapeutic effects are dependent on its intensity and duration (Bélair et al., 2018; Chan et al., 2019; Gronwald et al., 2018). High intensity or vigorous-intensity physical activity showed no positive effects in reducing depression and anxiety levels. Moreover in the past, the other viral outbreaks identified anxiety and depression persistent among individuals, thereby stating this concern should certainly not be neglected even in the present scenario. Individuals with mental health disorders show a lack of preference or a dislike to perform vigorous-intensity activities. Since mental health disorders add to the healthcare expenditure and burden, effective utilization of strategies to break physical inactivity by increasing time spent in light intensity activities may be the need of the hour. Especially in the post-COVID-19 era, physical inactivity would favourably report concern among both the symptomatic and asymptomatic individuals due to an overall decline in physical functioning. In such times, focusing on staying physically active by engaging in any form of physical activity or exclusively on the domestic and leisure-time physical activity may be simple yet effective strategies.
